# Neuralgia

**Published:** 1844-07-13

**Authors:** 


NEURALGIA.

Mr. Chippendale says he has tried the strong infusion of tobacco in frictions, in two cases of neuralgia, and in both of them met with complete success. He thinks an extract of tobacco mixed with cerate, would be more convenient for friction than the infusion.

